# Clinical efficacy and safety of sirolimus in childhood-onset systemic lupus erythematosus in real world

**DOI:** 10.1097/MD.0000000000031551

**Published:** 2022-11-18

**Authors:** Lei Zhang, Jie-Yuan Cui, Lin Zhang

**Affiliations:** a Department of Pediatric, The Third Hospital of Hebei Medical University, Shijiazhuang, China; b Department of Nephrology and Immunology, Children’s Hospital of Hebei Province, Shijiazhuang, China.

**Keywords:** cSLE, mTOR, prednisone, sirolimus, SLEDAI-2K

## Abstract

To investigate the effectiveness and safety of sirolimus in childhood-onset systemic lupus erythematosus in a real world. This is a retrospective real world clinical study. All childhood-onset systemic lupus erythematosus patients treated with sirolimus in Children’s Hospital of Hebei Province China were analyzed. They were treated with sirolimus and followed up regularly. The patients had systemic Lupus Erythematosus Disease Activity Index 2000 (SLEDAI-2K) score, levels of antidouble-stranded DNA antibody, complement components C3 and C4, 24-hour proteinuria and corticosteroid reduction were recorded at baseline and at 6, 12, and 18 months. Adverse events were also collected. Thirty-two patients were enrolled in the study. SLEDAI-2K were improved on all time-points (*P* < .05). Complement levels increased and the levels of antidouble-stranded DNA antibody decreased during treatment. The mean dose of prednisone tapered and achieved significant reduction after 12 months therapy (15.4 ± 5.8 mg/d to 4.8 ± 2.1 mg/d; *P* < .05). Sirolimus was well tolerated and only 5 patients (15.6%) experienced adverse events, all of which were classified as infections (2 bacterial infection and 3 viral infections). No deaths, severe infusion reactions, or hypersensitivity reactions were found. Sirolimus use was associated with a decrease in disease activity and ability to tolerate tapering of oral glucocorticoid dose with a favorable risk–benefit profile.

## 1. Introduction

Systemic lupus erythematosus (SLE) is a complicated chronic autoimmune disease that can result in multiple organ damage. The mortality of SLE has declined notably by reason of the utilization of glucocorticoids and development of immunosuppressive drugs in recent years.^[1]^ However, only one drug, belimumab, has been approved for SLE recently.^[2]^ Owing to its heterogeneity and great impact on quality of life, treatment options for SLE remain insufficient.

Mechanistic target of rapamycin (mTOR) is proved to be notably activated in the immune system in SLE patients.^[3]^ The mTOR inhibitor sirolimus (also called rapamycin) is reported to be effective in adult SLE with few side effects. Fernandez et al were the first to successfully use sirolimus in 9 patients with SLE who had failed treatment with other immunosuppressive drugs.^[4]^ The study by Yap et al focused on lupus nephritis (LN) and showed that sirolimus was effective in reducing 24-hour urinary protein as an induction therapy in active LN patients.^[5]^ However, In Peng’s study, proteinuria worsened in most LN patients.^[6]^ Recently, a phase I/II single-arm prospective clinical trial by Lai et al analyzed 29 patients with SLE without renal involvement. Their research shows that sirolimus can improve arthritis and rash and reduce SLE disease activity.^[7]^ Eriksson et al retrospectively summarize their long-term experience with sirolimus for the treatment of nonrenal manifestations of SLE and show that sirolimus is tolerable and safe over a nearly 4-year treatment period.^[8]^

Given that childhood lupus is not exactly the same as adult lupus. There have been few reports on the efficacy and safety of sirolimus in childhood onset SLE (cSLE). The aim of the present study was to describe the efficacy and safety of sirolimus in cSLE in a real-life setting in our department.

## 2. Methods

### 2.1. Patients

We reviewed the electronic medical records of the Children’s Hospital of Hebei Province China from March 2016 to March 2022. We identified a total of 32 cSLE patients. All cSLE patients(<18 yr) met the American College of Rheumatology in 1997 or the SLE classification standard revised by the American College of Rheumatology/Systemic Lupus International Collaborating Clinic (SLICC) in 2009. The baseline characteristics of all patients are shown in Table 1. All patients received oral sirolimus with a starting dose of 0.5 to 1 mg/m^2^ daily, and further titrated to maintain a therapeutic range of 5 to 10 ng/mL at least 6 months. Patients were followed up at our clinic at least 18 months after initial use of sirolimus. Before the treatment with sirolimus, all patients had systemic Lupus Erythematosus Disease Activity Index 2000 (SLEDAI-2K) ≥ 2 and had been treated with prednisone, hydroxychloroquine, and at least one immunosuppressant (Table 2). At the baseline of starting sirolimus, all patients were being treated with prednisone with a mean daily dose of 15.4 ± 5.8 mg/d, 30 patients with hydroxychloroquine, and 28 with one immunosuppressive drug.

**Table 1:** Baseline characteristics of the cSLE patients receiving sirolimus treatment.

	*N* = 32
Gender, female	27
Age at first infusion of sirolimus (yr)	11.4 ± 2.5
Clinical manifestations	5
Arthritis	19
Skin involvement	10
Oral ulcers	5
Serositis	8
Alopecia	12
Fever	14.92 ± 4.35
SLEDAI-2K score	15
Active nephritis	14
Laboratory features at baseline	22
Antidouble-stranded DNA antibody (positive)	19
Low levels of complement	
Leucopenia (< 4 × 10^9^/mL)	
Thrombocytopenia (< 150 × 10^9^/mL)	15

Data for categorical variables are expressed as *N* and those for continuous variables as mean ± SD.

cSLE = childhood-onset systemic lupus erythematosus.

**Table 2:** Baseline treatment with cSLE patients.

Baseline treatment	*N* = 32
Belimumab	14
Hydroxychloroquine	30
Prednisone	32
Mycophenolic acid	14
Cyclosporine	12
Leflunomide	2
Mean dose of prednisone (mg/d)	15.4 ± 5.8

Data for categorical variables are expressed as *N* and those for continuous variables as mean ± SD.

cSLE = childhood-onset systemic lupus erythematosus.

### 2.2. Assessments

Two pediatric rheumatologists were involved in the management of the cSLE patients over 18 months. Baseline information included demographics, clinical manifestations, laboratory results, and disease activity. Disease activity was assessed by SLEDAI-2K scores. Laboratory tests were done at each visit and included complete blood counts, urinalysis, 24-hour proteinuria (24hUPro) levels, liver and kidney function tests, and immunologic indices.

### 2.3. Statistical analyses

Continuous variables are expressed as mean ± standard deviation (SD) and categorical variables are expressed as frequencies (percentages). Analysis was performed using SPSS 21 (SPSS, Chicago, IL) and pictured in GraphPad Prism version 8.0 (Prism/Graphpad, Dan Diego, CA). Paired *t*-test was used to analyze the statistical significance of numerical variables and Chi-square or Fisher’s exact test was used to analyze the categorical variables before and after sirolimus therapy. Two-tailed *P*-value < .05 was considered significant.

## 3. Results

All patients had at least 2 points of SLEDAI-2K before use of sirolimus. A total of 23 (72%) patients were switched from other immunosuppressants to sirolimus (the patients either had an intolerance or a poor response to the previous immunosuppressants). Among 5 (16%) patients, sirolimus was added on without suspending previous immunosuppressants. Other 4 patients were directly added sirolimus on the premise of applying corticosteroids. The median time from onset of nephritis to initiation of sirolimus was 11 months.

Changes in clinical and serological parameters during sirolimus treatment at 6, 12 and 18 months are shown in Table 3. SLEDAI-2K improved over time at all time points. Complement levels increased during sirolimus treatment, antidouble-stranded DNA antibody levels decreased in the same way. Owing to this clinical and serological improvement, the mean dose of prednisone could be reduced from 15.4 ± 5.8 mg/d to 4.8 ± 2.1 mg/d at 12 months (*P* < .05) (Table 3). Overall, the number of patients treated with prednisone over 5 mg/day decreased from 26 (81%) at baseline to 11 (34%) at 18 months. The number of patients treated with prednisone up to 10 mg/day decreased from 15 (46%) at baseline to 2 (9%) at the last visit. Of note, the dose of prednisone could be reduced in all patients during the course. Four of them can be discontinued with prednisone. In addition, the dose of immunosuppressive agent could be reduced in 22 patients during the course, 3 patients could be discontinued after a mean period of sirolimus treatment of 10.8 ± 5.2 months.

**Table 3:** Changes in clinical laboratory indicators at the end of 6, 12, and 18 mo after sirolimus treatment.

	Baseline	6 mo	12 mo	18 mo
SLEDAI-2K	14.92 ± 4.37	4.56 ± 1.89*	1.73 ± 1.47*	2.21 ± 1.86*
C3 (g/L)	0.61 ± 0.12	0.97 ± 0.14*	1.22 ± 0.11*	1.19 ± 0.12*
C4 (g/L)	0.07 ± 0.03	0.17 ± 0.04*	0.21 ± 0.05*	0.19 ± 0.07*
Prednisone dose	15.4 ± d5.8	8.7 ± 2.3*	4.8 ± 2.1*	4.7 ± 3.3*

Data for continuous variables are presented as mean ± SD. C3, C4, complement components; SLEDAI-2K, Systemic Lupus Erythematosus Disease Activity Index 2000. Compared with baseline,

* *P* < .05.

The most common clinical manifestations that led to sirolimus use were low complement (87%) and cytopenia (75%). Sirolimus was discontinued in 3 patients owing to an inadequate response after 6 to 8 months treatment. In 2 of them, sirolimus was withdrawn owing to the development of LN (manifested by increased proteinuria and elevated serum creatinine). In the remaining one, sirolimus was withdrawn owing to the maintenance of cytopenia.

**Figure 1.**
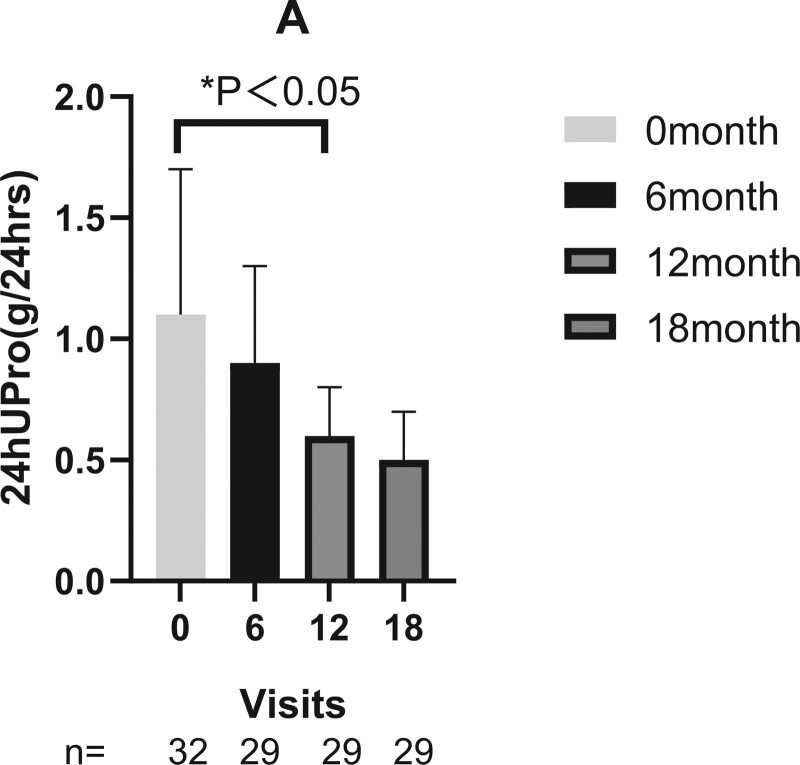
Efficacy of sirolimus on cSLE 24-hr urine protein at each time point. Sirolimus was discontinued in 3 patients owing to an inadequate response after 6 to 8 months treatment. *P < .05. cSLE = childhood-onset systemic lupus erythematosus.

All patients had a history of renal involvement. Two patients were LN Class V, 8 patient Class IV, 13 patients Class IV + V, and 9 patient Class III + V. The reduction in 24-hour proteinuria was statistically significant after 12 months sirolimus treatment (Fig. 1). There was no significant change in serum creatinine and eGFR of the children at each time point.

To determine safety, we reviewed the documented gastrointestinal side effects, such as mucositis, headaches, and infections that were attributed to sirolimus therapy. Finally, we only observed 5 patients as infections: 2 bacterial infection (one urinary tract infection and one lung infection) and 3 viral infections (2 patients had flu-like syndrome, 1 herpes zoster). All infections were relieved after treatment. Because of a high fever, the last patient was admitted to hospital and oseltamivir therapy was initiated, with recovery in a few days. Sirolimus and prednisone treatment were delayed for 14 days without signs of SLE flare. No deaths, severe infusion reactions, or hypersensitivity reactions were noted.

## 4. Discussion

Our study describes the effectiveness and safety of sirolimus retrospectively in cSLE patients in a real-life setting. Overall, sirolimus significantly reduced disease activity and serological indices in patients with cSLE.

mTOR is a serine threonine kinase that senses diverse environmental and intracellular signals including proinflammatory cytokines, growth factors and nutrients which involved in cell proliferation and metabolism.^[9]^ The mTOR pathway is also important in the activation of immune system and the reduction of inflammatory cytokine from macrophages.^[10]^ mTOR was involved in Th1 and Th17 cell differentiation, CD8 + memory cell survival, CD4 + CD25 + FoxP3 + Treg cell development and shifting from M1 macrophage phenotype to M2 phenotype.^[11–13]^ Due to the immunosuppressive effect of mTOR inhibitor, its clinical application was first in kidney transplantation. The immunosuppressive effect of mTOR inhibitor was first attributed to the inhibition of T cells and was further extended to B cells.^[14]^ Both B cells and T cells play critical roles in systemic lupus erythematosus. mTOR signaling activation was discovered in SLE and attracted attention as a therapeutic target.^[15]^ Rapamycin, a mTOR inhibitor, was first reported as a new therapeutic strategy for SLE in animal models and patients.^[4]^ Recent study demonstrated that rapamycin could improve disease activity in active SLE patients for 12 months treatment.^[7]^ However, there are fewer studies of sirolimus in cSLE.

In this article, we demonstrate that sirolimus is effective in reducing disease activity in cSLE. Our real-world study showed a significant reduction in SLEDAI-2K, further supporting the findings of previous studies. Furthermore, the improvement in serological activity, especially in C3 concentration, after sirolimus treatment was very significant. Our study showed normalization of low C3 levels in 60% of patients. Improvements with antidouble-stranded DNA antibodies were similar. These results are encouraging, and sirolimus holds promise as an alternative treatment option for SLE.

It was also helpful in the dosage tapering of prednisone. Clearly, in all of them, the dose of prednisone could be reduced, and in 4 of them the prednisone could be discontinued.

Evidence for sirolimus in SLE-related thrombocytopenia in previous studies was limited. However, many studies have demonstrated its efficacy and safety in immune thrombocytopenic purpura (ITP).^[16]^ In a randomized clinical trial, response rates in patients with ITP treated with prednisone plus sirolimus were similar to those treated with prednisone plus cyclosporine. (58% vs. 62%, *P* = .70).^[17]^ It is encouraging that the response rate of sirolimus in ITP patients is high even when used sirolimus alone. Both ITP and SLE have impaired Treg cells, which can suppress the secretion of cytokines and autoantibodies against platelets, as well as an increase in Th17 cells which can proinflammatory. Therefore, sirolimus could theoretically contribute to SLE-related thrombocytopenia. Our real-world study demonstrated a 50% CR rate for sirolimus in patients with SLE-associated thrombocytopenia. Two-thirds of patients achieved remission within 3 months and maintained stable platelet counts at subsequent visits. It is suggested that for SLE patients with thrombocytopenia, we can consider the use of sirolimus for treatment.

Regarding LN, evidence from lupus-prone mice supported the role of sirolimus in proteinuria. In one clinical research by Yap et al involved 16 patients with biopsy-proven LN, 3 out of 5 active LN achieved complete remission. Eleven patients with quiescent disease remained stable without renal flare.^[5]^ However, the role of sirolimus in LN proteinuria is illusive. In one study by Peng et al, 7 LN patients achieved remission of proteinuria, whereas 10 experienced worsening of proteinuria.^[6]^ In our study, the effect of sirolimus in proteinuria appears to be positive. Twenty-five patients achieved remission of proteinuria, and 6 patients experienced sustained microalbuminuria. Only one patient experienced worsening of proteinuria and serum creatinine. Unlike low dose sirolimus (1 mg/d) for LN adults in the Peng study, we strictly controlled the fluctuation of the plasma concentration of sirolimus at 5 to 10 ng/mL at first 6 months. Since it is beneficial to maintain the appropriate blood concentration of sirolimus in LN patients in our study, we believe that sufficient serum concentrations of sirolimus is necessary for therapy in LN.

In addition, at a relatively lower dose, sirolimus can also protect proximal tubular cells against damage from proteinuria, thus exerting its beneficial effects in proteinuric nephropathy. So, the use of low doses of sirolimus might be associated additional tubule interstitial protection. However, in Peng et al’s study, a very low dose (1 mg/d) of sirolimus may not be enough to control LN in adult SLE. Therefore, to our delight, maintenance of appropriate blood levels of sirolimus in patients with LN is necessary to preserve renal function. Moreover, more studies are required to determine a suitable dosage for the best application of sirolimus in child SLE with different manifestations.

All side effects of sirolimus in our study were mild and manageable. We only found infections as side effects and severe infections were not common when using sirolimus. A limitation of this study is that our study was only a retrospective study with no control group. Further randomized clinical trials should be designed.

## 5. Conclusion

In summary, maintain the proper blood concentration of sirolimus in cSLE patients were efficient in alleviating disease activity. Corticosteroids or immunosuppressive agent may be discontinued or significantly reduced in patients who maintain adequate sirolimus plasma concentrations. Severe side-effects were not seen. Only few patients discontinued sirolimus because of poor response. It is warranted that further randomized controlled trials evaluating the potential benefits of sirolimus in cSLE encompass larger number of cases.

## Acknowledgments

We would like to present our gratitude to the patients, nurses, and staff of the Rheumatology Department of Nephrology and Immunology, Children’s Hospital of Hebei Province who participated in this study.

## Author contributions

Lin Zhang responsible for the conception and design of the study. Jie-yuan Cui contributed to the acquisition of data. Lei Zhang and Jie-yuan Cui carried out the statistical analysis. Lei Zhang wrote the initial draft of the manuscript. All authors contributed to the conception or design of the work, or the acquisition, analysis, or interpretation of the data for the work; Lin Zhang revised the manuscript critically for important intellectual content and approved the final version for submission.

**Writing—original draft**: Lei Zhang.

**Formal analysis:** Jie-yuan Cui.

**Writing—review and editing:** Lin Zhang.

## References

[1] Ocampo-Piraquive V Nieto-Aristizábal I Cañas CA. Mortality in systemic lupus erythematosus: causes, predictors and interventions. Expert Rev Clin Immunol. 2018;14:1043–53. 30338717 10.1080/1744666X.2018.1538789

[2] Levy RA Gonzalez-Rivera T Khamashta M. 10 Years of belimumab experience: what have we learnt? Lupus. 2021;30:1705–21. 34238087 10.1177/09612033211028653 PMC8564244

[3] Suto T Karonitsch T. The immunobiology of mTOR in autoimmunity. J Autoimmun. 2020;110:102373. 31831256 10.1016/j.jaut.2019.102373

[4] Fernandez D Bonilla E Mirza N. Rapamycin reduces disease activity and normalizes T cell activation–induced calcium fluxing in patients with systemic lupus erythematosus. Arthritis Rheum. 2006;54:2983–8. 16947529 10.1002/art.22085 PMC4034146

[5] Yap DYH Tang C Chan GCW. Longterm data on sirolimus treatment in patients with lupus nephritis. J Rheumatol. 2018;45:1663–70. 30275264 10.3899/jrheum.180507

[6] Peng L Wu C Hong R. Clinical efficacy and safety of sirolimus in systemic lupus erythematosus: a real-world study and meta-analysis. Ther Adv Musculoskelet Dis. 2020;12:1759720X–20953336. 10.1177/1759720X20953336 PMC7493251 32973935

[7] Lai Z-W Kelly R Winans T. Sirolimus in patients with clinically active systemic lupus erythematosus resistant to, or intolerant of, conventional medications: a single-arm, open-label, phase 1/2 trial. Lancet. 2018;391:1186–96. 29551338 10.1016/S0140-6736(18)30485-9 PMC5891154

[8] Eriksson P Wallin P Sjöwall C. Clinical experience of sirolimus regarding efficacy and safety in systemic lupus erythematosus. Front Pharmacol. 2019;10:82. 30787878 10.3389/fphar.2019.00082 PMC6372521

[9] Liu GY Sabatini DM. mTOR at the nexus of nutrition, growth, ageing and disease. Nat Rev Mol Cell Biol. 2020;21:183–203. 31937935 10.1038/s41580-019-0199-y PMC7102936

[10] Gkirtzimanaki K Kabrani E Nikoleri D. IFNα impairs autophagic degradation of mtDNA promoting autoreactivity of SLE monocytes in a STING-dependent fashion. Cell Rep. 2018;25:921–933.e5. 30355498 10.1016/j.celrep.2018.09.001 PMC6218203

[11] Delgoffe GM Kole TP Zheng Y. The mTOR kinase differentially regulates effector and regulatory T cell lineage commitment. Immunity. 2009;30:832–44. 19538929 10.1016/j.immuni.2009.04.014 PMC2768135

[12] Mercalli A Calavita I Dugnani E. Rapamycin unbalances the polarization of human macrophages to M1. Immunology. 2013;140:179–90. 23710834 10.1111/imm.12126 PMC3784164

[13] Delgoffe GM Pollizzi KN Waickman AT. The kinase mTOR regulates the differentiation of helper T cells through the selective activation of signaling by mTORC1 and mTORC2. Nat Immunol. 2011;12:295–303. 21358638 10.1038/ni.2005 PMC3077821

[14] Zeng H Chi H. mTOR signaling in the differentiation and function of regulatory and effector T cells. Curr Opin Immunol. 2017;46:103–11. 28535458 10.1016/j.coi.2017.04.005 PMC5554750

[15] Perl A. Activation of mTOR (mechanistic target of rapamycin) in rheumatic diseases. Nat Rev Rheumatol. 2016;12:169–82. 26698023 10.1038/nrrheum.2015.172 PMC5314913

[16] Feng Y Xiao Y Yan H. Sirolimus as rescue therapy for refractory/relapsed immune thrombocytopenia: results of a single-center, prospective, single-arm study. Front Med (Lausanne). 2020;7:110. 32296709 10.3389/fmed.2020.00110 PMC7136762

[17] Li J Wang Z Dai L. Effects of rapamycin combined with low dose prednisone in patients with chronic immune thrombocytopenia. Clin Dev Immunol. 2013;2013:548085. 24363761 10.1155/2013/548085 PMC3865723

